# Endoscopic endonasal odontoidectomy for the treatment of basilar invagination

**DOI:** 10.3171/2020.3.FocusVid.20184

**Published:** 2020-07-01

**Authors:** Henry Ruiz-Garcia, Kelly Gassie, Lina Marenco-Hillembrand, Angela M. Donaldson, Kaisorn L. Chaichana

**Affiliations:** 1 Department of Neurological Surgery, Mayo Clinic, Jacksonville, Florida;; 2 Department of Otolaryngology. Mayo Clinic, Jacksonville, Florida

**Keywords:** odontoidectomy, basilar invagination, endoscopic

## Abstract

Basilar invagination is a challenging dilemma that neurosurgeons may face. Herein, we present a case of a 65-year-old female with a history of rheumatoid arthritis and status post a previous C4–7 ACDF who presented to our clinic with progressive weakness in her bilateral upper and lower extremities. Imaging revealed basilar invagination. She underwent an endoscopic endonasal odontoidectomy followed by an occiput–C6 fusion. We present the endonasal portion of the procedure and have highlighted the technical nuances of this approach. Our goal is to provide better insight into this surgical strategy when dealing with basilar invagination.

The video can be found here: https://youtu.be/aeMbvI_zYQA

## Transcript

In this video we will discuss the endonasal endoscopic approach used to perform an odontoidectomy in a patient with basilar invagination. The relevant anatomy and technical nuances will also be presented.

**0:33** Case illustration. This is the case of a 65-year-old woman who presented with progressive quadriparesis. She started her symptoms with bilateral upper extremities weakness 6 months ago, and this progressed until she was wheelchair bound. She was not able to perform any ADL at the time of her visit. She also presented with dysphagia and urinary incontinence that progressed over the past month.

In the physical exam, the patient presented with limited neck mobility and midthoracic sensory level. Her motor strength was severely diminished in her upper extremities and her interossei muscle were completely atrophic and nonfunctional, with diminished lower extremities strength, specially proximally.

Her relevant PMH was positive for rheumatoid arthritis and cervical stenosis for which she underwent C4–7 ACDF approximately 10 years ago.

The preoperative imaging of the CT scan in the sagittal view shows the presence of basilar invagination with calcification of the CVJ ligaments. The MRI also confirmed the presence of cervical spinal cord compression and the presence of basilar invagination.

In this 3D-rendered image, this is an anterior view showing the clivus, the arch of C1 and C2, and the right and left ICAs that are relevant during the preoperative planning period, as they run bilateral to the anterior tubercle of C1, which will be drilled during surgery (Alfieri et al., 2002). The posterior view of this 3D-rendered image shows the invagination of the dens and its relationship with the right and left vertebral arteries, which are also important to consider preoperatively.

Based on the patient’s presentation as well as the imaging, a surgical plan was made which should be a two-stage approach. In the first approach, we would conduct an anterior resection of the odontoid through the nasal cavity, followed by a stage 2 approach which would include a posterior cervical decompression and occiput–C6 fusion. We spaced these procedures over 2 days where the first stage was done in one day, followed by the second stage on another day.

**2:46** Anatomical considerations. In regard to some anatomical considerations, the palatine line, which is in green, is very helpful to determine the feasibility of the endonasal approach (de Almeida et al., 2009), where a line is drawn in a sagittal view, along the plane of the hard palate in the direction of the CVJ. If this lesion is located above this line, the endonasal endoscopic approach is preferred, as the working corridor is above the hard palate (Hussain et al., 2018; Liu et al., 2015).

The nasoaxial line is helpful in determining the inferior limit of the approach, where this is drawn from the midpoint, between the rhinion and the anterior nasal spine, toward the posterior edge of the hard palate and the CVJ. This line has been described as more predictive than the nasopalatine line, as the latter may overestimate the inferior limit of this approach (Liu et al., 2015).

Here we see different potential anterior approaches to the CVJ. Lesions extending beyond the nasoaxial line may benefit from a transoral approach (Ponce-Gomez et al., 2014).

**3:45** Patient position. During the first stage, the patient was positioned supine and the head was fixed with three-point Mayfield head holders in neutral position. The surgical navigation was used to help identify the clivus, C1, and C2, before and during the procedure.

**4:02** Exposure of nasopharyngeal fascia. This procedure was done in combination with our rhinology colleagues. Lidocaine/epinephrine was injected in the nasal septum as well as in the inferior turbinates bilaterally to minimize blood flow from the sphenopalatine artery. Septoplasty was performed, and a low posterior septectomy was taken superiorly until it was flushed with the roof of the choana.

The middle and superior turbinates were then lateralized bilaterally, and both middle turbinates were fractured to expand our access. After all these were performed and hemostasis was achieved, we could clearly identify the posterior pharyngeal wall.

Here we can appreciate the surface anatomy of the posterior nasopharynx (Alfieri et al., 2002), where we can identify the posterior wall, the bilateral choanas, the Eustachian tubes, and the soft palate.

The carotid arteries were identified based on navigation as well as Doppler investigation, and then we started to obtain exposure of the lower third of the clivus by making an inverted U-shaped incision in the posterior pharyngeal wall using an extended bovie tip that was protected. The bovie dissected the mucosa at the posterior nasopharynx as well as went through the muscular bed of the longus colli in order to get down to the basisphenoidal fascia. The flap was elevated until the area just inferior to the soft palate using bovie as well as a suction bovie; a red 8-0 rubber catheter was used to achieve retraction of the soft palate in order to allow expanded inferior access below the hard palate. The surgical navigation was then used to confirm the safety of the surgical corridor in regard to the vascular supply. We then started the dissection in order to identify the inferior clivus and the C1 anterior arch. We first dissected above C1, and then we drilled over it using a high-speed drill and the ultrasonic aspirator. We then kept drilling on the C1 anterior arch going deeper and superior until the dens was identified; as our drilling and dissection took place laterally, we were cognizant of both internal carotid arteries located lateral to our dissection. As our dissection and drilling plane took us deeper, we found more calcified soft tissue between the C1 anterior tubercle and inferior aspect of the dens that we needed to flush and remove in order to achieve adequate decompression. After we drilled the calcification, we started to dissect superiorly to move the bottom of the clivus. We extended the dissection along the C1 anterior tubercle more laterally; we then identified the C2 dens and started to approach it. The dens was then drilled internally until it was eggshelled in a canoe formation. In patients with inflammatory process such as rheumatoid arthritis, it may be difficult to remove C2 from the tentorial ligaments as they may be adherent because of the inflammation. We therefore needed to proceed very carefully in order to minimize the risk of a cerebrospinal fluid leak. As the drilling and dissection took place superiorly and inferiorly from our normal angle of view, a 30° scope would help visualize both the upward and downward directions. Round dissector as well as a straight dish dissector may be used to remove the dens from the surrounding craniovertebral ligaments. The dens can then be out fractured laterally and the bone brought down to the surgical field. After this was done, no bone was identified at the top of the dens as well as along the area that was parallel with the inferior aspect of C1, so we proceeded with the closure.

**8:44** Closure. It was irrigated with vancomycin-impregnated saline fluid; fat was obtained from the lower quadrant of the abdomen and placed in the epidural plane as this area then to protect the dura. The U-shaped mucosal flap was then reapproximated; in this case there was a defect that originated at the top of the flap from the muscle contraction, and in these cases a new free mucosal flap can be obtained from the middle turbinate to close this defect. The area was reinforced with Tisseel glue and Gelfoam to complete the final closure.

**9:22** Results. Postoperative CT showed good removal of the anterior portion of the dens with good decompression anteriorly. This x-ray shows the occipital–C6 fusion that was performed the following day. During this approach, the patient was carefully placed prone on a Jackson table and Mayfield pins, a midline incision was then made from the inion to C6, and the posterior elements were exposed. Decompression was then done from the inferior aspect of the foramen magnum to C3; following this and using stereotactic navigation, screws were placed in C2 parts bilaterally. Bilateral mass screws were then placed from C3 to C6 using anatomical landmarks, an occipital plate was then placed, the screws were connected with rods, and fusion was supplemented with BMP [bone morphogenic protein], iliac crest bone grafts, and demineralized bone matrix.

There were no complications and the patient had a significant improvement in upper and lower extremities. She was discharged to a rehabilitation facility on postoperative day 5. At 4-month follow-up, her strength had improved, and the patient was able to perform many ADL and transfers independently.
